# Rich cities, poor countryside? Social structure of the poor and poverty risks in urban and rural places in an affluent country

**DOI:** 10.1177/02690942221104774

**Published:** 2022-06-05

**Authors:** Oliver Hümbelin, Lukas Hobi, Robert Fluder

**Affiliations:** 69477 Bern University of Applied Sciences, Department of Social Work, Switzerland

**Keywords:** poverty, poverty risk factors, regional difference, admin-data, random forest

## Abstract

This paper contributes to the field of regional poverty literature by using linked tax data to examine poverty in a large district in Switzerland with one million inhabitants and rural and urban parts. We measure poverty using income and asset-based approaches. Our regional comparison of the social structure of the poor shows that poor people in rural areas are more likely to be of retirement age. Among the workforce, the share of poor is larger for those who work in agriculture compared to those working in industry or the service sector. In urban areas, the poor are more often freelancers and people of foreign origin. Despite where they live, people with little education, single parents, and people working in gastronomy/tourism are disproportionately often poor. We then use a random forest based variable importance assessment to clarify whether the importance of poverty risks factors differs in urban and rural locations. It shows little regional differences among the major poverty risk factors, and it demonstrates that the opportunity structure, like density of workplaces or aggravated access in mountain areas, seem to be of minor importance compared to risk factors that relate to the immediate social situation.

## Introduction

Ending poverty is a priority for the global community and the top objective in the Sustainable Development Goals (SDGs). Good data is key on this journey to combat poverty. As pointed out by Atkinson (2019: 105), “we will not be able to reach our goal unless we have data to show whether or not people are actually lifting themselves out of poverty. Collecting good data is one of the most powerful tools to end poverty.” This statement could be seen as less important for European countries, which all have national statistics that allow to observe and study the development of poverty. Indeed, the data infrastructure in Europe is quite good compared to many other parts of the world, but most countries lack the options to disaggregate their statistics on relevant subnational levels. Therefore, estimating regional poverty rates, given the current data resources, is a difficult task (Copus et al., 2015). At the same time, there is a substantial amount of literature highlighting the importance of regional aspects of poverty or place-based approaches to poverty that are likely important in a diverse region such as Europe (Bentley and Pugalis 2014; Copus et al., 2015; Madanipour and Weck, 2015; Madanipour et al., 2015). To develop the adequate poverty policies necessary to achieve the poverty goals of the agenda by 2030 and beyond, it is crucial to understand how regional economic structure, and places in general, contribute to people living below the poverty line (Zhou und Liu, 2019).

Our study contributes to this discussion in two ways. First, from the perspective of using data to improve the knowledge of poverty in wealthy countries, this study demonstrates how linked administrative data can be used to investigate poverty. While the use of administrative data to develop policies is not new (Hotz et al., 1998), novel ways to apply them have emerged. Of special importance for distributional studies are tax data (Hümbelin and Farys, 2016). This kind of data is indispensable for inequality studies, particularly in regards to top-incomes (Alvaredo et al., 2013; Piketty, 2014), but it also has hurdles, and simultaneously huge potential, when studying the lower part of the distribution. We linked tax data to other administrative data and survey data to overcome these hurdles and to build a rich dataset that allows to study poverty. Throughout this paper, we refer to poverty as having insufficient financial resources to meet the national line of minimal livelihood. We acknowledge that poverty, especially in affluent countries, must be broadly understood through concepts such as social exclusion (Madanipour et al., 2015), multidimensional poverty (Alkire and Apablaza, 2016), and nonmonetary indicators (Atkinson, 2019). However, it is still common to operationalize poverty by measuring financial resources (UN, 2017). This has the advantage of measuring poverty in a conceptional, clear manner, and many studies show that the lack of financial resources is at the core of poverty. As Switzerland is one of the few countries that levy taxes on wealth, our data includes detailed information on this factor. Thus, we can measure poverty by assessing the financial situation of a household by its income and its wealth (Brandolini et al., 2010). Furthermore, because households are geocoded to identify residential municipalities and the data cover the population region-wide, this has great potential for the study of regional differences.

Second, the current study respectively investigates poverty on subnational levels for small areas by performing a case study for the canton of Bern in Switzerland. It is a region with one million inhabitants, 352 municipalities and with large urban and rural areas. This can delineate poverty patterns within and between urban and rural areas in a comparatively prosperous region of the world. These results are relevant to other parts of Europe because the structural change of the economy that leads to different outcomes for cities and the countryside can be found there likewise. Economic life in cities increasingly happens in the service and knowledge-oriented tertiary sector, while the importance of primary and partly secondary sector economic activities, like agricultural and industrial work, declined but is still present in the countryside. Recently, with the strong impact of digitalization on the economy, ICT-driven economic advances seem to result in strong economic growth, especially in urban areas. Meanwhile, rural areas are struggling to attract high-skill workers, and the rift between regions deepens (Eckert et al., 2019). Since solid data for small area regional analysis is still scarce, it is not well studied to what extent these macro-level changes translate to poverty risks from a regional perspective. To gain more insight into the differences and similarities of poverty between urban and rural parts, we analyze the following research questions:

1. *Are people living in cities or the countryside more at risk of becoming poor?*
2. *Are different social groups poor in cities versus the countryside?*
3. *How important is the opportunity structure with respect to the risk of being poor?*
4. *Have commonly known poverty risk factors been judged as equally important in urban and rural locations?*

To answer these questions, we first demonstrate how our study complements the literature on regional poverty in affluent countries. We then describe the data and the indicators used to measure poverty and related risk factors and present our strategy for analysis. We then present the results on how the social structure of the poor differs in cities and the countryside. The analysis is extended with a random forest based variable importance risks assessment for individual and regional characteristics. This approach is also used to assess differences in poverty risk factors for cities versus the countryside.

## Theory

### Poverty in affluent countries and the role of a spatial approach to poverty

#### Between economic opportunities and welfare mitigation

To understand the reasons behind poverty, it is necessary to distinguish between micro- and macro-level factors as well as the interaction between the two levels (Sidney, 2009). Some studies point out that individual and/or household level characteristics, including employment status, educational level, citizenship, health status, and the civil state, are strongly associated with the risk of becoming poor (Atkinson and Da Voudi, 2000; Atkinson et al., 2004). Some argue that differences across race, citizenship, gender, or age groups may relate to discrimination within a society (Hümbelin and Fritschi, 2018; Tilly, 1999). However, whether these characteristics lead to poverty is strongly shaped by the macro-level context such as economic change, shifts in the demands of the labor market, and alterations in social security provision by the state, especially the scope of and access to social benefits.

On the one hand, researchers investigate structural re-configurations across Europe since the 1970s (Andreotti et al., 2012). Deindustrialization, the upheaval of the global economy, technological change and its impact on local labor markets have undoubtedly changed which occupations are profitable. In this vein, it seems that technological change dampened median wage income growth and increased polarization of the wage distribution and skill premiums in several high-income countries (Katz and Autor, 1999; Katz and Margo, 2014). As Eckert et al. (2019) argue, these developments led to a new urban bias in economic growth that bears the risk of exacerbating the divide between cities and the countryside.

On the other hand, researchers point out that the level of *decommodification, state redistribution or, more generally, the social protection scheme and its ameliorative interventions* shape how well and who is protected by social security (Esping-Andersen, 1999; Vandecasteele, 2015). While all Western welfare states currently provide social security, they struggle to adapt to new social risks (Bonoli, 2007) such as precarious employment, long-term unemployment, and working poverty (Crettaz, 2013). At the same time, Causa and Hermansen (2020) found that the redistributive effect declined on average from 1995 to 2014 and across most of the OECD-countries. As suggested by the growing literature on non-take-up of social benefits, this tightening of welfare tools also negatively impacted the poor’s access to social benefits (Eurofound, 2015; Hernanz et al., 2004; Lucas et al., 2021).

#### Relevance of places

While there is a lively discourse on the role of macro-level factors as described above, other research focuses on regional patterns of poverty. According to Zhou and Liu (2019) and the geography of poverty, it is essential to understand the spatial pattern of poverty. It is acknowledged that this approach has the potential to deepen our understanding on how (and why) people in rich countries get poor (Bentley and Pugalis, 2014; Madanipour and Weck, 2015), and it offers a promising link between the micro- and the macro-level. It is, however, also commonly known that estimating regional poverty rates, given the available data, is a challenging task (see Copus et al., 2015). Thus, empirical studies following the local environmental approach are still scarce, and a holistic theory combing macro-, meso-, and micro-level dynamics still needs to be developed. To that end, the following paragraphs synthesize the literature to highlight what is needed to further that goal.

A recent report from the World Bank noted that people in the bottom 40% of the income distribution disproportionally often live in rural areas (World Bank, 2020). Worldwide rurality seems to be an area with a heightened poverty risk. But is this true for a generally prosperous region like Europe? Michálek and Výbošťok (2019) recently studied the development across all 28 EU member states and found that in general economic growth helped to decrease poverty but in most EU countries, only a minority benefited from economic growth resulting in increased inequality and often an increase of poverty. According to the authors, it is therefore crucial to understand the unequal distribution of wealth within countries to allow tackling poverty. In this regard, understanding regional differences might deliver key elements for policy makers. Indeed urbanity, or rurality, are often associated with poverty in different ways. According to the European Commission (2010), poverty is more common in densely populated areas than in less populated counterparts in the EU-15 countries. This “urban exclusion” is mainly an effect of the segregation of the poor who tend to live gathered in affordable neighborhoods (Foulkes and Schafft, 2010; Madanipour, 2015). However, there is also poverty in rural places (Bertolini et al., 2008). This is mostly attributed to sectoral change stemming from technological progress and a decline of employment opportunities in specific areas. This might be a decline of the industrial sector (Bennett et al., 2000) or the agricultural sector (Shucksmith and Schafft, 2012). While some leave the area, others remain trapped by their lack of opportunity and mobility. All in all, it seems that change in economic structure does not translate in a simple way to changes in poverty risks in cities and the countryside. The reasons behind this paradigm must be clarified to determine whether poverty risks differ in cities and the countryside.

Blank (2005) offers a promising way to further develop the relationship between macro- and micro-levels by introducing clear statements on how regional features might influence poverty risks:

1. One key role is played by the *natural environment.* It defines accessibility or ease of travel from a specific location. Moreover, climate and natural resources define possible economic activities. After all, cities were established with a favorable initial situation with respect to their natural environment. Accordingly, Liu et al. (2021) found that accessibility of a region plays a major role in identifying poor regions in China.
2. The *economic structure* refers to the labor demand generated by the local economy. Is it an industrial or agricultural area? Or is labor demand about specialized tech or services? This influences job opportunities, income growth, or the risk of having unsteady revenues.
3. *Public institutions* are those organizations operating within an area to ensure its functioning. They include general infrastructure, such as the police or the educational system, as well as institutions providing public assistance programs. These institutions represent the welfare regime and are—together with the economic structure—part of the opportunity structure of inhabitants of a specific region. More recently, Baker (2020) showed that politics and more specifically power resources are a major source of regional inequalities and argued that this is the main reason why the south of the US is poorer than other regions.
4. *Social norms or expectations* are shaped by local institutions and commonly shared behavior and experiences. They set an informal bound of rules and are often a component of the discussion of welfare stigma usage (Moffitt, 1983). More isolated and rural communities may have stronger social norms. These norms and general trust in the form of social capital can be a resource or an obstacle to overcome poverty as Harrison et al. (2019) argue finding that social capital and poverty are strongly correlated in US counties.
5. The last factor refers to *demographic characteristics.* Locations with only low skilled workers often have high shares of poor people. While demographic characteristics are easily measured, they provide limited causal information. However, because they are correlated with specific behavioral issues, they provide useful signals about what types of policies are needed and useful.

So far, it has been demonstrated that labor market characteristics are essential, that these characteristics are shaped by the regional economic structure, and that this structure is influenced by the natural environment in turn. Moreover, social security instruments are relevant, and accessibility can differ across regions due to differences in public institutions and social norms. While these concepts help highlight areas that need further attention, it is not conclusively clarified which characteristics of local labor markets and occupation structure are associated with high poverty risks or which groups protected by social security are threatened to fall through the safety net. Copus et al. (2015) offered further insights into what type of regional economic structure is associated with increased poverty risk by estimating regional at-risk-of-poverty rates for 20 European countries. They also performed a correlation analysis to identify potential socioeconomic drivers of poverty, where they synthesize several theoretical strands of regional poverty drivers. Their results showed a strong correlation between at-risk-of-poverty rates and the unemployment rate as well as employment shares in elementary occupations like cleaners or agricultural tasks. They also found evidence to suggest that welfare regimes moderate these risks. However, correlated macro-indexes can be difficult to interpret since they bear the risk of ecological fallacy. Further studies combing rich individual and contextual features may be a promising way to further our understanding.

Despite these studies, it remains unclear how important, if at all, the spatial poverty approach is, and, which aspect is most relevant. This question must be addressed by further research. Therefore, we combine an inductive and deductive research approach to find regional patterns in the data based on characteristics already identified in the general poverty literature. To gain an initial understanding, we study demographic profiles of the poor in cities and the countryside. Next, we assess poverty risk factors on the individual and at the level of the opportunity structure to unravel which features of the opportunity structure are important compared to the individual level. Finally, we study if commonly known risk factors are associated with similar poverty risk importance across regions.

#### Poverty and the welfare state in Switzerland

To understand the following results, it is important to know where Switzerland is placed regarding its poverty prevalence and protection. Although Switzerland is one of the richest countries worldwide, a considerable part of the population lives with a disposable household income below the national poverty line. Official statistics collected by the SDG country report show (Swiss Confederation, 2018) that poverty rates in Switzerland rose slightly recently. In 2016, 7.5% of Switzerland’s permanent resident population—around 615,000 people—were affected by income poverty. According to official numbers, poverty rates are particularly high for retirees as well as non-European immigrants, people with low education levels, single parents, and for people living in a household with poor or no access to the labor market (FSO, 2020).

Concerning the welfare regime in the terminology of Esping-Andersen (1990), Switzerland is classified today as a rather classic Western European welfare state with mixed elements (Armingeon, 2001). Yet, Ebbinghaus (2012) points out that a nationwide classification falls short, because Switzerland is organized in a federal way with great autonomy at the level of cantons, which are the main substate divisions in Switzerland. Armingeon et al. (2004) classify all Swiss cantons according to the principles of Esping-Andersen, showing heterogeneity regarding taxation and the instruments addressing poverty.^1^ While social insurances addressing unemployment, old age, disability, or maternity are regulated nationwide, the instruments addressing poverty differ. While social assistance, the last safety net, is guided by national recommendations, cantons can adjust as they see fit. For further means-tested benefits, like health-premium subsidies and family supplementary benefits, the scope of action of the cantons is large. Finally, access to social benefits is organized on communal levels, which can provide more or less support and information (Hümbelin, 2019). Since poverty policies are strongly driven by cantons, it is a known deficit that cantonal or even communal poverty rates are unavailable.

## Data and methods

### Measuring poverty using linked tax data

Many European poverty studies and official statistics on poverty use survey data, for example, the European Union Statistics on Income and Living Conditions. However, survey data insufficiently represent the low income population (Häder et al. 2012; Korinek et al., 2006), which can only partly be corrected with statistical weights. In contrast, tax data provide a more reliable approach to study the financial situation. For our analyses, we use linked fiscal and administrative data called WiSiER data (Wanner, 2019) for the year 2015 for the canton of Bern^2^—a large canton of Switzerland with a mix of urban and rural parts, representing the situation in Switzerland quite well. The restrictions of the dataset, and definitions of main variables, closely follow Fluder et al. (2020). The data with complete information represents 89% of the permanent population (*N* = 910,346 persons, 428,709 households).^3^ We define households as people sharing the same residential unit, based on the register of buildings and apartments. As means-tested benefits are not taxable, tax data do not contain them. Therefore, we link information of social benefits to our data (i.e., social assistance, reduction of health insurance premiums, supplementary benefits to old age, survivor’s, and the disability insurance). Our linked tax data allows us to reliably assess financial poverty. Moreover, the nearly complete representation of the population permits detailed analyses on the level of socioeconomic groups and small-scale spatial regions.

For our analyses, we measure poverty as a lack of financial resources. A first indicator measures income poverty—referring to the social subsistence level of Switzerland and the official absolute poverty line. Accordingly, a household is poor when its expenses for the minimum needs (as set by national standards, see BKSE, 2020; Schweizerische Konferenz für Sozialhilfe, 2015) outweights its total income, including social transfers from insurances and other benefits. However, this indicator incompletely describes the financial situation since households may have financial reserves to supplement their resources from income. Thus, we build a second indicator using an asset-based poverty measurement approach (Brandolini et al., 2010; UN, 2017). According to this indicator, a household is poor if it does not have sufficient income to finance the daily needs and does not have enough reserves to cover the needs for 12 months. Since household members generally share resources, we assess poverty at the household level. However, the unit of analysis is the individual.

To assess poverty risk of individuals from different social groups and in different social situations, we need information beyond those on financial resources. We build these variables only partly from the register data, and we use further information from the structural survey, a large survey complementing register data in Switzerland. Since the structural survey is a sample survey, this information is available only for a subsample of the dataset. Therefore, for analyses using the structural survey (especially the variable importance analysis), the sample is reduced from 910,346 to 106,850^4^ observations. The subsample differs from the overall population in its main characteristics to some extent. Thus, we balanced our subsample using e-balance^5^ (Athey and Imbens, 2017; Hainmueller, 2012) to achieve a representation of the overall population with respect to the distribution of age groups, gender, nationality, marital status, household type, and income classes. Furthermore, we linked information on level of individuals with municipality profiles from the FSO for the year 2015 (FSO, 2015) to gain information for several measures of the opportunity structure as described in the next section.

### Poverty risk factors and the opportunity structure

We distinguish between three groups of characteristics with potential influence on individual poverty risk as shown in Table 1. For the social structural analysis, we distinguish between features of *social groups* and *social situation*. Our third set of variables measures the *opportunity structure*. A detailed table including descriptive statistics can be found in the Appendix.

**Table 1.** Indicators used by group of variables.

Social groups	Social situation		Opportunity structure (municipality level)	
Indicator name	Indicator name	Description	Indicator name	Description
Age groups	Civil state		Density of workplaces	Workplaces by population
Citizenship	Education	Highest completed education	Economic sector of workplaces	Dominant activity in primary, secondary, or tertiary sector
Sex	Employment status hh	Dependent or independently working, non-working, or retired	Employees by economic sector	Dominant activity in primary, secondary, or tertiary sector
	Health status	Degree of disability according to social insurance agencies	Language	Dominant language spoken
	Household size		Mountain area	
	Household type		Percentage of unemployed	
	Number of employed hh members		Political leaning	Share of votes for social party minus share of votes for popular party
	Sector of occupation		Population share of employed	
			Urbanity	Cities, Agglomerations, and Municipalities on the countryside

To differentiate between *social groups* we use age groups, sex, and citizenship. These characteristics mostly remain stable throughout one’s lifetime as age groups refer to cohorts with a common historical-biographical background and citizenship influenced by birthplace. Drawing on Tilly (1999), we argue that an effect related to these characteristics can indicate discrimination. To represent different *social situations* people live in, we use a set of variables often found in the literature to study poverty risks. These variables include education, household type, civil state, sector of occupation, health status/degree of disability, employment status, number of employed household members, and household size. These factors describe the immediate social situation, for example, access to the labor market or skills (education, sector of occupation), and they can be addressed by poverty interventions (Nielsen et al., 2008).

Following Blank (2005) and Copus et al. (2015), we identify different aspects of the *opportunity structure*. To capture *accessibility of a region by its natural environment,* we distinguish mountain and non-mountain areas. To address the *economic structure*, we use variables dividing regions based on predominantly agricultural, industrial, and service activities. We also delineate based on reported employees and on workplaces. Additionally, we measure the regional performance of the labor market based on the density of workplaces and the population share of employed and unemployed. Finally, we include indicators measuring *public and community institutions and social norms*. We use political leaning as a proxy for both dimensions. Since local welfare provision is organized by local communities and welfare ideologies strongly differ by right and left wing ideologies (Roosma et al., 2016), it is reasonable to build an indicator capturing the political leaning of a municipality based on parliamentary voting. Moreover, we built an indicator of the predominant language. Since language regions depict different cultures and traditions that represent one of the dimensions of the political cleavage system in Switzerland (Linder et al., 2008), we substitute part of the different institutional and normative settings with this variable.

As a general proxy for spatial differences, we use the urbanity variable based on official classification of municipalities. It classifies every municipality as either a city, being in the agglomeration, or the countryside. This last variable is also used in the empirical part to distinguish urban and rural parts.

### Determining variable importance using random forest

Similar to Liu et al. (2021), we use random forest to assess the importance of a variable empirically. Random forest is a machine-learning approach increasingly gaining attention in applied economics that combines large sets of classification and regression trees (Athey, 2019; Nosratabadi et al., 2020). Random forest has the advantage of accounting for non-linear relationships and interactions in the data without the need to explicitly know and specify them (Athey and Imbens, 2017; Molina and Garip, 2019). Since we study individual, household, and contextual characteristics which are expected to interact with each other, this is an important feature. Finally, random forest allows to relatively quantify the importance of a variable. Importance in this context refers to an increase in the probability of correctly identifying poor people that can be attributed to a specific variable.

Since continuous variables could gain more consideration in the variable importance analysis than categorical variables (Strobl and Zeileis, 2008), we recoded all our regressors as categorical variables.

We started with a basic model that fitted a logit regression with asset-based poverty and income poverty as binary outcomes and included all independent variables. By running a variance inflation factor analysis (O’brien, 2007), we ensured that collinear factors were excluded (Tables 5 and 6 in the Appendix). We then fitted a random forest model with income poverty and asset-based poverty as binary outcomes and included all independent variables. Covariates were tested to find the best covariate to split the data into two portions, minimizing the number of wrongly classified observations, until only a small number of observations was left at the endpoints (“leaves”). To balance the high sensitivity of trees to small changes in the data, random forest combines a large number of trees to a “forest.” To achieve a decorrelated set of trees, two random components are present in the algorithm: (1) each tree is fit to a randomly resampled part of the original data where each subsample is taken with replacement and (2) for each split, only a random subset of the covariates is tested.^6^

Although the model has difficulty correctly classifying the poor, it has good overall performance, with only 4.12% overall classification error rate for income poverty and 2.67% for asset-based poverty (Table 2).

**Table 2.** Confusion matrices for random forest models.

Confusion matrix for rf model on asset-based poverty				Confusion matrix for rf model on income poverty			
	True	True			True	True	
Classified =	0	1	Class. error rate	Classified =	0	1	Class. error rate
0	101’134	277	0.27	0	99’450	439	0.44
1	2’515	509	83.17	1	3’865	670	85.23
overall			2.67	overall			4.12

Since variable importance is a relative measure depending on how well an indicator can be predicted and asset-based poverty can be predicted better than income poverty, variable importance will be lower for asset-based poverty. To enable comparisons across the two indicators, we standardized variable importance by dividing the variable importance of each regressor by the total variable importance of all regressors in each model.

## Results

### Social structure of the poor in cities, agglomerations, and in the countryside

**Figure 1.**
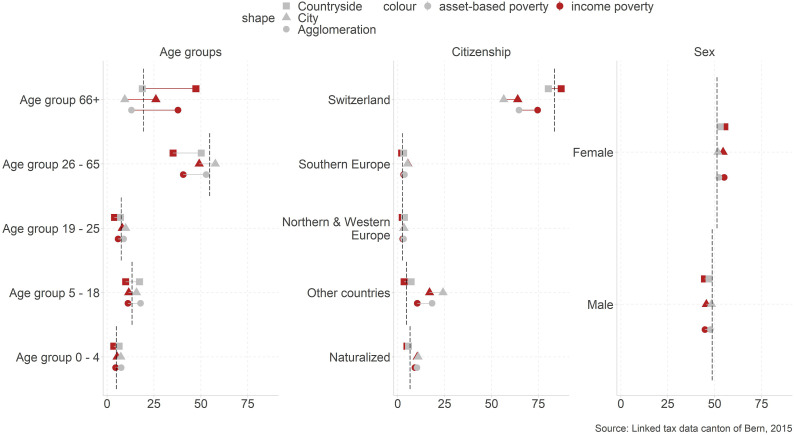
Social structure by social groups. Note. The figure shows the composition of the poor by age group, nationality, and gender for the poor living in urban, metropolitan, and rural areas. The dashed line shows the composition of the total population*.*

First, we observe how the population of the poor differs by comparing them to the overall population (dashed line). We begin with the characteristics we use to distinguish social groups (Figure 1). To determine the spatial dimension, we divide the analysis by the population living in cities, the countryside and agglomerations. The analysis is done using the income and the asset-based poverty measure. Data-visualizations demonstrate the general patterns. Related numbers and further analysis showing population shares and poverty rates by urbanity for all variables can be found in Hümbelin et al. (2021).

From a *methodical perspective*, we observe that results differ if we use the income or the asset-based poverty measure. Differences are very strong regarding age groups, but they are also present for citizenship. Especially, the share of poor retirees drops significantly when switching from income poverty (38.9% of all poor) to the asset-based poverty indicator (13.9%). Since pension plans in Switzerland partially rely on private savings and people have the option to get capital from their occupational pensions instead of a rent, it is integral to the Swiss pension plan that retirees in Switzerland partly live off their financial reserves. It is therefore not surprising that less retirees fall below the asset-based poverty line when including financial reserves. Regarding citizenship, results demonstrate that Swiss citizens are better able to build financial reserves than foreign citizens from countries other than South, North, or Western Europe.

Regarding *spatial dimensions,* specific patterns can be identified. First, the share of poor retirees and poor Swiss people in general is higher in the countryside than in other regions. Second, poor people of working age and foreigners are found more often in cities and agglomerations than in the countryside. However, the associated increased poverty risk is not found among all migration groups. There are hardly any differences between foreigners from Northern and Western Europe and Swiss nationals. Migrants from Southern Europe are, by contrast, overrepresented among the poor in cities and migrants from other countries are overrepresented among the poor in cities and agglomerations. Among this latter group are many migrants from former Yugoslavia who immigrated to Switzerland in the 1990s, but also from numerous other Asian, American, or African nations. Surprisingly, naturalized people are overrepresented among the poor meaning that this group still has an increased risk of becoming poor, but it is significantly reduced compared to foreigners that have not (yet) obtained a Swiss passport. Previous literature focused more on within-country migration movements, for example, from rural to urban parts and how this influences poverty rates (c.f. Foulkes and Schafft, 2010). The elevated prevalence of income and asset-based poverty for migrants from outside of Northern, Western, and Southern Europe and naturalized citizens is however novel.

From an *overall perspective,* we find the following interesting patterns. Gender differences are rather small. While women are over-proportionately more often poor than men (55.5% female) regarding income poverty, these differences get smaller when including assets, although a difference remains (52.3%). Regarding age groups, we identify a dramatic shift of the poverty profile. When assessing poverty with the classic income poverty measure, retirees dominate the numbers. In contrast, assessing poverty while including financial reserves changes the focus to children and families. Then, these groups are disproportionately often poor and lack financial reserves to handle episodes of income poverty. This is possibly a consequence of people using any financial reserves for the purchase of housing in the phase of starting a family. Financial reserves in later phases of life are therefore more common.

**Figure 2.**
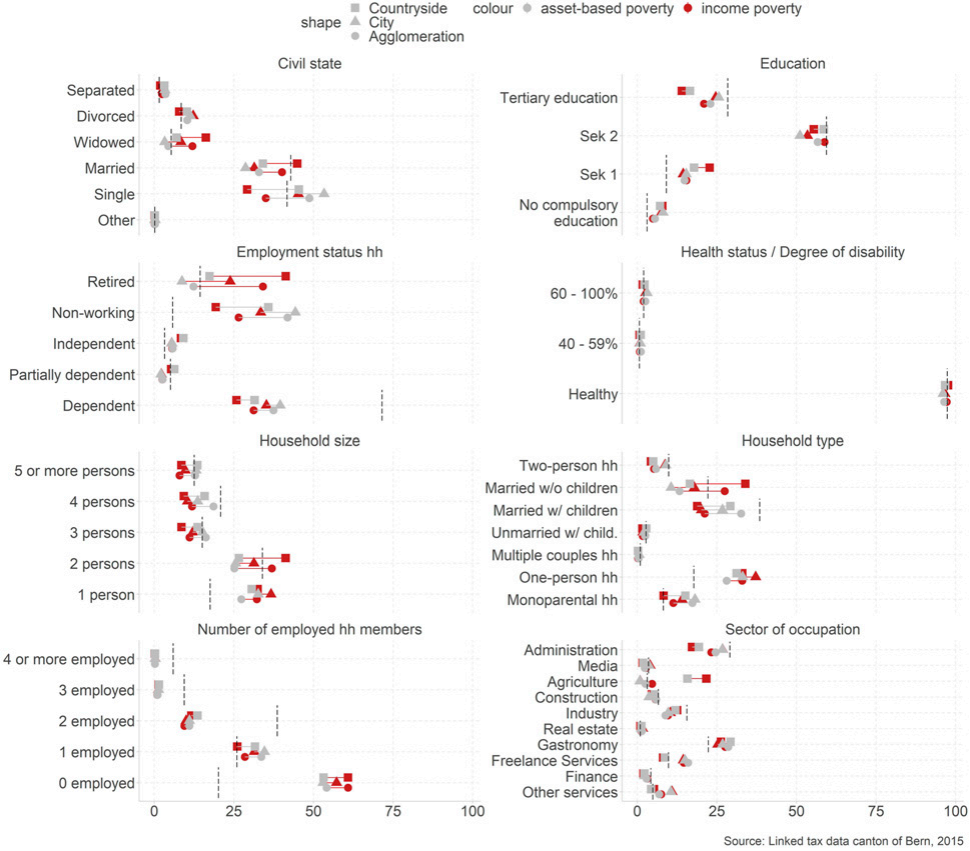
Social structure by social situation. Note. The figure shows the composition of the poor across civil state, education, employment status, health status/degree of disability, household size, household type, number of employed household members and sector of occupation for poor living in cities, agglomerations, and in the countryside. The dashed line shows the composition of the total population.

We also show differences regarding several characteristics that define the *social situation* (Figure 2). When focusing on the asset-based poverty measure as a lead indicator, we find many *overall patterns* that are in line with the poverty literature. Poor tend to be more often singles in one person (16.6% in the poverty population vs 8.1% in the overall population) or monoparental-households (30.9% vs 17.8%). They are slightly over-proportionally more often divorced or separated (10.6% vs 8.4%). They tend to have lower education (no compulsory education: 7.1% vs 3.1%), and they are more often not or weakly attached to the labor market (non-working: 40.5% vs 5.7%). We also see differences regarding the sector of occupation—which links the individual situation and the opportunity structure. Overall, we see that poor work comparatively less often in administration (23.6% vs 29.1%), industries (10.2% vs 15.5%), and finances (2.4% vs 4.2%), and more often in the gastronomy (28.1% vs 22.3%). Regarding health status, we observe no striking anomalies, signifying that those qualifying for a disability rent are not disproportionally often among the poor. However, we cannot show how health status affects poverty risks beyond disability status.

While these patterns are supported regarding the *spatial dimension*, we observed some interesting spatial differences. In the countryside, we more often find poor widowed, retirees, and people with low education. We also see more poor that are working independently or in the agricultural industry. In cities, the poor are more often singles. In agglomerations, married with children are disproportionally often poor. Non-working poor are also more dominant in cities. Regarding the sector of occupation, poor are over-proportionally often freelancers and working in small services like hairdressers or private household helpers.

From the *methodical perspective,* we see again the big change between income and asset-based poverty assessment for retirees (34.5% income based vs 12.9% asset-based). We also see a shift for civil state. While the drop of the share of poor for widowed probably mirrors the situation of retirees, there is also a shift regarding married and single persons. This shift may refer to different life stages and possibilities to accumulate financial reserves.

### Relevance of the opportunity structure

**Figure 3.**
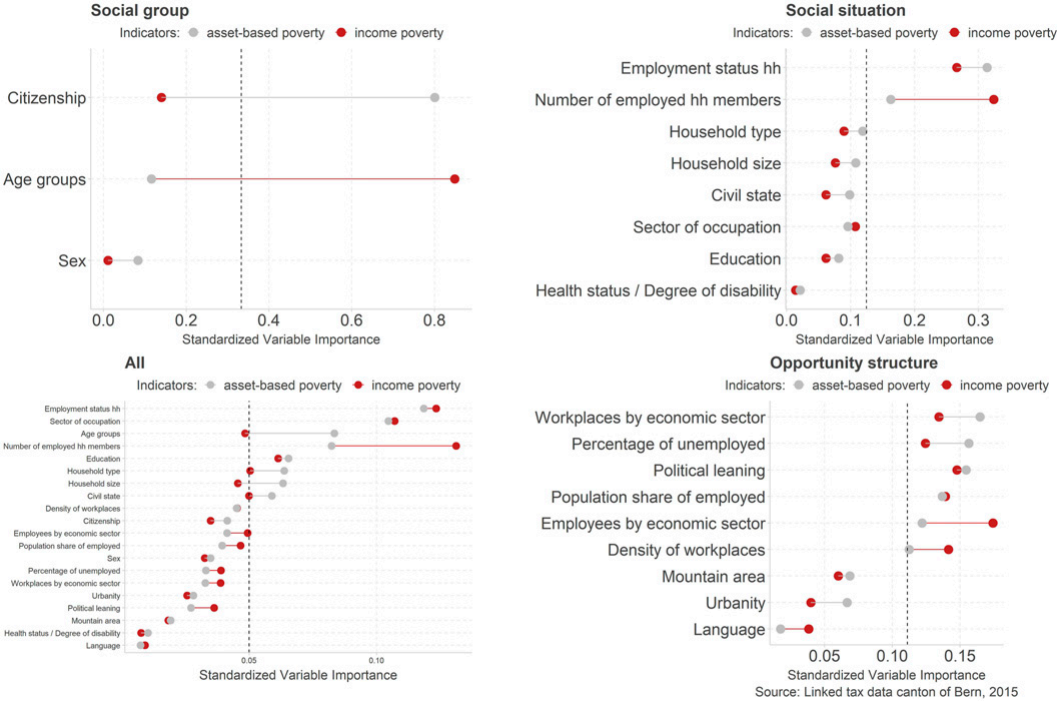
Variable importance by social group, social situation, and opportunity structure. Note. The figure shows standardized variable importance to predict poverty derived from random forest models. The dashed line represents average variable importance across all variables. Variables are ordered by importance to predict asset-based poverty*.*

Moving forward, we evaluate the importance of different variables to distinguish the relevance of the characteristics for social groups, social situation, and the opportunity structure. We show results for all *social group*, *social situation*, and the *opportunity structure* characteristics separately as well as a model including all variables (Figure 3).

Overall*,* the variables capturing the social situation are the most important when predicting poverty. The ranking is led by characteristics that measure a household’s attachment to the labor market, closely followed by the variable that measures the sector of occupation. While this last variable is less important in the analysis covering only characteristics of the social situation, it gains importance in the full model because of its interaction with the variables measuring the opportunity structure. At the same time, it can be seen in the full model that all variables measuring the opportunity structure on the level of municipalities are clearly less important for predicting poverty, suggesting that the opportunity structure is less important compared to the social situation. Among the social group variables, the age group is the most dominant variable (in the full model) while gender and citizenship are of minor importance if other variables are included in the model.

While the share of poor based on the asset-based poverty measure is slightly higher in cities compared to poverty rates in agglomerations and in the countryside,^7^ the mere differentiation of these three types of urbanity has comparatively low predicting power to identify poverty (see urbanity). Among the characteristics that measure the *opportunity structure,* those that stand for economic structure are more important than the variables measuring accessibility (mountain area) and the institutional and normative context (political leaning and language).

### Regional differences of poverty risk factors importance

To determine if poverty risk factors vary by regions, we ran three separate random forest models for people living in cities, agglomerations, and in the countryside.

**Figure 4.**
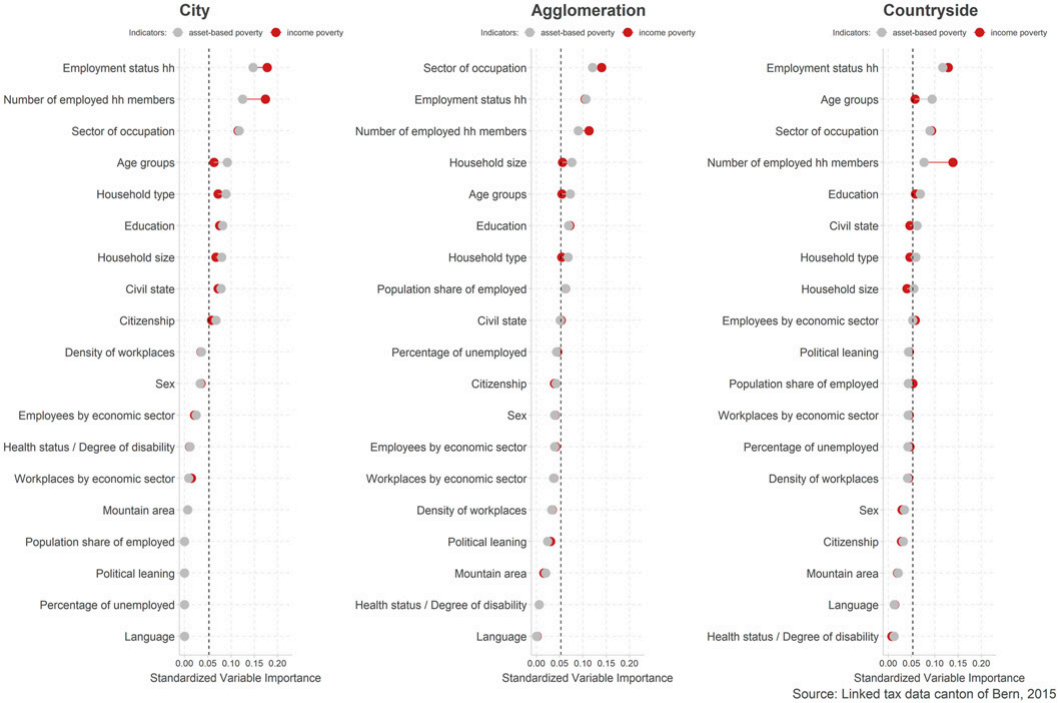
Importance of poverty risk factors in cities, agglomerations and in the countryside. Note. The figure shows standardized variable importance to predict poverty derived from random forest models. The dashed line represents average variable importance across all variables. Variables are ordered by importance to predict asset-based poverty.

Results in Figure 4 suggest that the importance of poverty risk factors is rather constant across regions. Irrespective of where people live, the employment status, the number of household members that are active in gainful employment and the sector of occupation, remain the most important to predict poverty. Similarly, characteristics of the social groups, such as sex and citizenship but with the exception of age group, are of subordinate importance in all regions. Poverty at different life stages ranks higher generally, but it seems to be a topic that is of special relevance in the countryside, where the importance of the age groups variable takes second place. Finally, the opportunity structure seems to have comparatively lower importance in all three urbanity types.

## Summary and discussion

Following theories referring to the economic change that favors urban areas with its service and tech-based economies (Eckert et al., 2019), it might be assumed that poverty occurs mainly in disconnected areas on the countryside (Shucksmith und Schafft, 2012). This is not true, at least in Switzerland in our area of study. Even though income poverty is more prevalent in the countryside, leading to higher income poverty rates in rural parts compared to cities, this is a methodical artifact. Since we can construct an asset-based poverty measure accounting for household incomes and financial reserves, we can show that some defined as poor based on income do not qualify once the wealth situation is taken into consideration. This affects regional poverty rates through the different social structure in cities and in the countryside. On the one hand, the share of income poor with financial reserves is higher among self-employed and Swiss citizens. These groups are disproportionally more often present in the countryside. On the other hand, income poverty without financial reserves is more common among people with poor access to the labor market and foreigners from outside of Europe. These groups again are relatively more prevalent in cities. In the end, if we assess poverty rates with the asset-based approach, we find that rates are highest in cities (7.0%), then the countryside (5.0%), and they are lowest in the agglomerations (4.4%). Still poor people live in cities and in rural parts alike. As our study shows, there are several ways in which a place-based approach to poverty leads to a better understanding of how regional opportunities link to poverty.

While our study confirms general findings in the poverty literature like that the poor disproportionally often have low education or are single parents (Atkinson et al., 2004) or that they are working in unsteady occupations with seasonal fluctuations like gastronomy or tourism, our analysis is also able to show that poverty has different faces in cities and in rural parts:

1. In *rural parts,* the poor are more often likely to be retirees. Although the number of poor retirees drop drastically if financial reserves are accounted for, poor retirees are more prevalent in rural compared to urban parts. As retirees are equally present in urban and rural parts, this is a result of an increased risk of being poor, as the age specific poverty rates for individuals aged 66 and above show: in cities 4.2% are poor while 6.6% are in the countryside. We assume that the non-take-up of social benefits that is more prevalent in rural regions (Hümbelin, 2019) plays a part in explaining this difference. Keeping in mind that our asset-based measure is constructed to give a general sense of the importance of financial reserves and includes reserves for only 1 year, it can be assumed that more retirees are at risk of poverty. Further studies should make use of the asset-based poverty approach and take a closer look at the financial reserves of retirees. It could be estimated if retirees’ reserves are sufficient to maintain a standard of living above the poverty line until they reach the usual life expectancy. Thus, it could be assessed how many are at risk of old-age poverty. A second noticeable group of poor in rural parts are those who work in agriculture. Although Switzerland is known as a country with comprehensive subsidies for the agricultural sector, not every farmer is able to adapt to the changing conditions of global markets. It seems that some of this group are threatened to be left behind as part of economic and societal change, as already made clear by Shucksmith and Schafft (2012).
2. In *urban parts,* other groups are disproportionally often among the poor. Freelancers, cultural professionals, and people working in small personal services like housekeepers can more often be found among the poor. While the new tech-based economy leads to innovations and hubs mainly in cities (Eckert et al., 2019), this does not necessarily imply that everyone profits from these developments. It also illustrates that freelancers take higher risks and, therefore, possibly slip more often below the poverty line compared to occupations with more consistent working conditions like those working in industries, finances, or administration. Also, a special phenomenon of cities is the greater portion of poor foreigners. This group is clearly more often present in cities than in rural parts. 44.5% of the poor in cities are non-Swiss, while in the countryside only 19.7% of the poor are foreigners (22.5% is the overall share of non-Swiss inhabitants). However, not every region of origin bears a heightened risk of poverty. Foreigners with a background outside of Europe are by far more strongly affected than foreigners from European neighbor countries. Foreigners from the northern or western part of Europe are not excessively represented among the poor. This highlights that global migration in a wealthy country like Switzerland is associated with different poverty risks for different groups of foreigners. Highly qualified migrants from countries with a similar cultural background as Switzerland do not necessarily experience heightened poverty risks, unlike migrants with a background outside of Europe without professional training or skills that do not necessarily fit the demands of the Swiss labor market. The heightened poverty prevalence we discover suggests that these groups can struggle to take a foothold even if they are allowed to stay in Switzerland. Since we measure poverty based on incomes post all means-tested benefits, this result also reflects that these groups encounter hurdles to apply for social assistance. Indeed, the receipt of social assistance can become a reason for a withdrawal of the residence permit, which has the effect that these groups are incentivized to not apply for social assistance and makes them an especially vulnerable group.

All in all, our study confirms previous findings showing that activity in basic occupations like cleaning or agricultural tasks are associated with higher risks of poverty (Copus et al., 2015). Furthermore, it presents findings that raise attention for others risk groups as well. Freelancers, cultural professionals, and foreigners, that are mainly found in urban areas are also confronted with an increased poverty risk. Poor retirees seem to be a specificity of rural parts. Based on these findings, it is recommended to study the living conditions of those groups in detail and to ensure poverty programs are set up to address their specific situations. In rural parts, it should be ensured that there are counseling programs addressing the situation of retirees and farmers. Poverty policies in cities should have a special focus on people with unsteady working conditions such as freelancers and those engaged in small personal services. Additionally, special programs should be tailored to reach out to foreigners with a non-European background.

Our machine-learning based risk factor assessment suggests that the immediate social situation, such as not having access to gainful employment and the sector of occupation, are the most dominant factors predicting poverty. Other characteristics of the *social situation,* like household type, marital status, and education, are also important to predict poverty. Overall, the *social situation* variables outperform the characteristics that describe *social groups*, such as citizenship^8^ and gender, except for age groups. In that case, it also ranks highly in the importance ordering, especially in the rural part. It seems that poverty at different life stages, especially in the countryside, is a topic worth further research. Finally, all characteristics that relate to the *opportunity structure* have lower predictive power compared to the social situation and social groups. This does not necessarily mean that factors like accessibility (Liu et al., 2021), social norms, local economic structure, and local institutions (Blank, 2005) do not play any role. On the contrary, we believe such factors are crucial elements in a holistic approach to addressing and theorizing about poverty. Although the region we study is quite heterogenous with respect to regional economic structure, urbanity, and political orientation, it is still a well-developed area with solid infrastructure that allows people to commute within the area. Basic coverage of social service institutions is also provided. Moreover, since we study the situation in one canton, the basic welfare regulations are constant across the whole region. To gain more insights with respect to the relevance of the opportunity structure, we recommend comparative studies between different regions of the same country, comparative studies between different countries or studies with a longitudinal approach that enables researchers to study changes in the opportunity structure.

Our detailed analysis of the social structure of the poor as well as the risk factor analysis provide valuable insights into which spatial dimensions relate to the poverty phenomena in an affluent country. Furthermore, it lays the groundwork for further causal analysis that can delve into the “why” behind the observed patterns.

## Notes

1. The canton we use for our empirical analysis is the canton of Bern. It is described as a canton with liberal and conservative welfare elements. In contrast to other cantons, it has no special poverty instruments other than the ones defined by national law.

2. To date, it is not possible to obtain tax data for all Swiss Cantons because of legal reasons.

3. Individuals with incomplete information are excluded. This includes persons living in collective households such as nursing homes, persons with missing household data, or those who are not taxed under the regular procedures, such as pending tax cases or foreigners who are taxed in other ways.

4. Using data pooled over the years 2010–2016 for the structural survey (Wanner, 2019).

5. All computations were done in R (R Core Team, 2021). Ebalance was calculated using the package *ebal* version 0.1–6 (Hainmueller, 2012).

6. Random forest models were calculated using the package *ranger* version 0.12.1 (Wright and Ziegler, 2017). In our analysis, we used 500 trees (num.trees) for each random forest model. We used the (rounded down) square root of the number of covariates to try at each split (mtry) in our analysis.

7. The income-based poverty rate is highest in the countryside 11.5%, followed by cities 10.4% and agglomerations 8.2%. With the asset-based poverty indicator the ranking changes to the following: 7.0% cities, 5.0% countryside, and 4.4% agglomerations.

8. This is not a contradiction to the findings above. Since the results of the risk factor analysis assess the importance of a single factor in the context of all variables at hand, the result here just identifies the dominant factors. Since some groups of foreigners are disproportionally often among those not having sufficient access to the labor market, the result of the risk factor assessment signifies that it is the access to the labor market that affects the poverty risk, not citizenship.

## Appendix

**Table 3.** Population shares by indicator (*N* = 910,346).

Indicator name | *Desc*	Indicator categories	Pop. shares, %	Source | Theme
Age groups	0–4	4.9	Statpop Social groups
	5–18	13.3	
	19–25	7.5	
	26–65	54.7	
	66+	19.5	
Citizenship Naturalized if country of origin is not Switzerland but nationality is	Switzerland	83.5	Statpop Social groups
	Northern and western Europe	2.6	
	Southern Europe	2.7	
	Other countries	4.6	
	Naturalized	6.6	
Sex	Female	51.4	Statpop Social groups
	Male	48.7	
Civil state	Single	41.7	Statpop Social situation
	Married	42.9	
	Widowed	5.3	
	Divorced	8.4	
	Separated	1.6	
	Other	0.2	
Education Highest completed education	No compulsory education	3.1	Structural survey Social situation
	Sek 1	9.1	
	Sek 2	59.4	
	Tertiary education	28.4	
	Data not available	66.7	
Employment status hh^a^	Dependent	71.5	Fiscal data Social situation
	Partially independent	5.1	
	Independent	3.2	
	Non-working	5.7	
	Retired	14.4	
Health status Degree of disability	Healthy	97.4	OASI data Social situation
	40–59%	0.7	
	60–100%	2	
Household size	1 person	17.5	Statpop Social situation
	2 persons	34	
	3 persons	15.1	
	4 persons	20.9	
	5 or more persons	12.6	
Household type	One-person hh	17.8	Statpop Social situation
	Married w/o children	22.2	
	Two-person hh	9.8	
	Monoparental hh	8.1	
	Married w/children	38.5	
	Unmarried w/children	2.7	
	Multiple couples hh	0.9	
Number of employed hh members	0 employed	20.2	Fiscal data Social situation
	1 employed	25.9	
	2 employed	38.6	
	3 employed	9.4	
	4 or more employed	5.9	
Sector of occupation	Agriculture	3.1	Structural survey Social situation
	Industry	15.5	
	Construction	6.6	
	Gastronomy	22.3	
	Media	3.5	
	Finance	4.2	
	Real estate	0.9	
	Freelance services	9.8	
	Administration	29.1	
	Other services	4.9	
	Data not available	88.2	

^a^Dependent if share of indep. hh earned inc. < 0.2. Partially dependent if < 0.8. Independent if ≥ 0.8. Non-working if no hh earned inc. and no member age ≥ 65. Retired if no hh earned inc. and min. 1 member age ≥ 65.

**Table 4.** Pop. shares by indicator – Opportunity structure (*N* = 910,346, Data source = Munic. profiles).

Indicator name | *Desc*	Indicator categories	Pop. shares, %	Distribution
Density of workplaces^a^ Ratio of workplaces over population	1st quartile [0.040–0.064]	31	Min: 0.040 Max: 0.275 Median: 0.074
	2nd quartile [0.064–0.078]	32.9	
	3rd quartile [0.078–0.102]	16.4	
	4th quartile [0.102–0.275]	19.8	
Economic sector of workplaces If share of workplaces in sector higher than in median of communities	Predominantly agricultural	7.3	
	Pre. agricultural and industrial	9	
	Pre. industrial	2.3	
	Pre. agricultural and services	1.3	
	Pre. industrial and services	26.5	
	Pre. services	53.6	
Employees by economic sector If share of employees in sector higher than in median of communities	Predominantly agricultural	4.8	
	Pre. agricultural and industrial	7.2	
	Pre. industrial	7.0	
	Pre. agricultural and services	4.2	
	Pre. Industrial and services	43.5	
	Pre. services	33.4	
Language	German	94.8	
	French	5.2	
Mountain area	Non-mountain area	77.6	
	Mountain area	22.4	
Percentage of unemployed^a^	1st quartile [0.3%–0.9%]	5.1	Min: 0.3% Max: 9.5% Median: 1.3%
	2nd quartile [0.9%–1.3%]	15.6	
	3rd quartile [1.3%–1.8%]	28.1	
	4th quartile [1.8%–9.5%]	51.2	
Political leaning^a^ Share of votes for social party minus share of votes for popular party	Right	8.6	Min: −83.7% Max: 21.9% Median: −12.9%
	Center-right	11	
	Middle	18.8	
	Center-left	61.6	
	Missing	0.2	
Population share of employed^a^ Ratio of employed over population	1st quartile [0.101–0.261]	11.2	Min: 0.101 Max: 2.235 Median: 0.396
	2nd quartile [0.261–0.364]	13.9	
	3rd quartile [0.365–0.491]	21.4	
	4th quartile [0.494–2.235]	53.5	
Urbanity Based on official classification	City	25.7	
	Agglomeration	35.8	
	Countryside	38.5	

^a^If share is ≤ 1st quartile/median/3rd quartile of municipalities or higher.

**Table 5.** Variance inflation factor analysis for logit model on asset-based poverty.^a^

Indicator name	GVIF	Degrees of freedom (Df)	Adjusted GVIF^b^	Indicator name	GVIF	Degrees of freedom (Df)	Adjusted GVIF^b^
Age groups	10.5	3	1.48	Sector of occupation	1.46	9	1.02
Citizenship	1.28	4	1.03	Density of workplaces	7.92	3	1.41
Sex	1.27	1	1.13	Economic sector of workplaces	16.6	5	1.32
Civil state	9.88	5	1.26	Employees by economic sector	17.3	5	1.33
Education	2.45	3	1.16	Language	2.45	1	1.57
Employment status hh	43.3	4	1.6	Mountain area	2.53	1	1.59
Health status/Degree of disability	1.03	2	1.01	Percentage of unemployed	4.92	3	1.3
Household size	495.39	4	2.17	Political leaning	5.89	3	1.34
Household type	1307.91	6	1.82	Population share of employed	3.08	3	1.21
Number of employed hh members	70.8	4	1.7	Urbanity	11.4	2	1.84

GVIF: Generalized Variance Inflation Factor. ^a^All variables were kept in the set of regressors, since no variable exceeded the upper bound of 2.23 for the adjusted GVIF, which is a commonly used rule of thumb for categorical variables.

^b^GVIF^(1/(2*Df)).

**Table 6.** Variance inflation factor analysis for logit model on income poverty.^a^

Indicator name	GVIF	Degrees of freedom (Df)	Adjusted GVIF^b^	Indicator name	GVIF	Degrees of freedom (Df)	Adjusted GVIF^b^
Age groups	16.4	3	1.59	Sector of occupation	1.71	9	1.03
Citizenship	1.39	4	1.04	Density of workplaces	8.99	3	1.44
Sex	1.22	1	1.11	Economic sector of workplaces	21.8	5	1.36
Civil state	9.22	5	1.25	Employees by economic sector	21.6	5	1.36
Education	1.97	3	1.12	Language	2.83	1	1.68
Employment status hh	79.1	4	1.73	Mountain area	2.79	1	1.67
Health status/Degree of disability	1.05	2	1.01	Percentage of unemployed	5.5	3	1.33
Household size	292.42	4	2.03	Political leaning	6.91	3	1.38
Household type	952.50	6	1.77	Population share of employed	3.33	3	1.22
Number of employed hh members	30.1	4	1.53	Urbanity	13.6	2	1.92

GVIF: Generalized Variance Inflation Factor. ^a^All variables were kept in the set of regressors, since no variable exceeded the upper bound of 2.23 for the adjusted GVIF, which is a commonly used rule of thumb for categorical variables.

^b^GVIF^(1/(2*Df)).

## References

[bibr1-02690942221104774] Alkire S Apablaza M. (2016) Multidimensional Poverty in Europe 2006–2012: Illustrating a Methodology. Oxford Poverty and Human Development Initiative Working Papers. Available at: https://ora.ox.ac.uk/objects/uuid:e28d3bc0-a74a-4034-a333-f3fc09fe8c2f. Last accessed on May 24th, 2022.

[bibr3-02690942221104774] Alvaredo F Atkinson AB Piketty T , et al. (2013) The top 1 percent in international and historical perspective. Journal of Economic Perspectives 27(3): 3–20.

[bibr4-02690942221104774] Andreotti E Mingione E Polizzi E (2012) Local welfare systems: a challenge for social cohesion. Urban Studies 49(9): 1925–1940. DOI: 10.1177/0042098012444884 10.1177/0042098012444884

[bibr5-02690942221104774] Armingeon K (2001) Institutionalising the Swiss welfare state. West European Politics 24(2): 145–168. DOI: 10.1080/01402380108425437 10.1080/01402380108425437

[bibr6-02690942221104774] Armingeon K Bertozzi F Bonoli G (2004) Swiss worlds of welfare. West European Politics 27(1): 20–44. DOI: 10.1080/01402380412331280793 10.1080/01402380412331280793

[bibr7-02690942221104774] Athey S (2019) The Impact of Machine Learning on Economics. The Economics of Artificial Intelligence. Chicago, IL: University of Chicago Press. Available at: https://www.degruyter.com/document/doi/10.7208/9780226613475-023/html. Last accessed on May 24th, 2022.

[bibr8-02690942221104774] Athey S Imbens GW (2017) The state of applied econometrics: causality and policy evaluation. Journal of Economic Perspectives 31(2): 3–32. DOI: 10.1257/jep.31.2.3 10.1257/jep.31.2.329465214

[bibr9-02690942221104774] Atkinson AB (2019) Measuring Poverty Around the World. Princeton, NJ: Princeton University Press.

[bibr10-02690942221104774] Atkinson AB Marlier E Nolan B (2004) Indicators and targets for social inclusion in the European Union. JCMS: Journal of Common Market Studies 42(1): 47–75. DOI: 10.1111/j.0021-9886.2004.00476.x 10.1111/j.0021-9886.2004.00476.x

[bibr11-02690942221104774] Atkinson R Da Voudi S (2000) The concept of social exclusion in the European Union: context, development and possibilities. JCMS: Journal of Common Market Studies 38(3): 427–448. DOI: 10.1111/1468-5965.00229 10.1111/1468-5965.00229

[bibr12-02690942221104774] Baker RS (2020) Why is the American south poorer? Social Forces 99(1): 126–154. DOI: 10.1093/sf/soz149 10.1093/sf/soz149

[bibr13-02690942221104774] Bennett K Beynon H Hudson R (2000) Coalfields Regeneration: Dealing with the Consequences of Industrial Decline. Bristol, UK: Policy Press.

[bibr14-02690942221104774] Bentley G Pugalis L (2014) Shifting paradigms: people-centred models, active regional development, space-blind policies and place-based approaches. Local Economy 29(4–5): 283–294. DOI: 10.1177/0269094214541355 10.1177/0269094214541355

[bibr15-02690942221104774] Bertolini P Montanari M Peragine V (2008) Poverty and Social Exclusion in Rural Areas. Final Study Report.

[bibr16-02690942221104774] BKSE (2020) Handbuch Sozialhilfe – Stichwort Grundbedarf für den Lebensunterhalt (GBL). Available at: http://handbuch.bernerkonferenz.ch/stichwoerter/stichwort/detail/grundbedarf-fuer-den-lebensunterhalt-gbl/. Last accessed on May 24th, 2022.

[bibr17-02690942221104774] Blank RM (2005) Poverty, policy, and place: how poverty and policies to alleviate poverty are shaped by local characteristics. International Regional Science Review 28(4): 441–464. DOI: 10.1177/0160017605278999 10.1177/0160017605278999

[bibr18-02690942221104774] Bonoli G (2007) Time matters postindustrialization, new social risks, and welfare state adaptation in advanced industrial democracies. Comparative Political Studies 40(5): 495–520.

[bibr19-02690942221104774] Brandolini A Magri S Smeeding TM (2010) Asset-based measurement of poverty. Journal of Policy Analysis and Management 29(2): 267–284. DOI: 10.1002/pam.20491 10.1002/pam.20491

[bibr20-02690942221104774] Causa O Hermansen M (2020)Income redistribution through taxes and transfers across OECD countries, research on economic inequality. In Rodríguez JG Bishop JA (ed) Inequality, Redistribution and Mobility. Bingley, UK: Emerald Publishing Limited, Vol. 28, 29–74. DOI: 10.1108/S1049-258520200000028002 10.1108/S1049-258520200000028002

[bibr21-02690942221104774] Copus A Melo PC Kaup S , et al. (2015) Regional poverty mapping in Europe – challenges, advances, benefits and limitations. Local Economy 30(7): 742–764. DOI: 10.1177/0269094215601958 10.1177/0269094215601958

[bibr22-02690942221104774] Crettaz E (2013) A state-of-the-art review of working poverty in advanced economies: theoretical models, measurement issues and risk groups. Journal of European Social Policy 23(4): 347–362. DOI: 10.1177/0958928713507470 10.1177/0958928713507470

[bibr23-02690942221104774] Ebbinghaus B (2012) Comparing welfare state regimes: are typologies an ideal or realistic strategy. In: Draft Paper presented at European Social Policy Analysis Network, ESPAnet ConferenceBd, Edinburgh, UK, 6–8 September 2012, p. 120.

[bibr24-02690942221104774] Eckert F Ganapati S Walsh C (2019) Skilled Scalable Services: The New Urban Bias in Economic Growth. Preprint. Institute Working Paper. DOI: 10.21034/iwp.25 10.21034/iwp.25

[bibr25-02690942221104774] Esping-Andersen G (1990) The Three Worlds of Welfare Capitalism. Cambridge, UK: Polity Press.

[bibr26-02690942221104774] Esping-Andersen G (1999) Social Foundation of Post-Industrial Societies. Oxford, UK: Oxford University Press.

[bibr27-02690942221104774] Eurofound (2015) Access to Social Benefits: Reducing Non-take-up. Luxembourg: Publications Office of the European Commission.

[bibr28-02690942221104774] European Commission (2010) Investing in Europe’s Future:Fifth Report on Economic, Social and Territorial Cohesion. Luxembourg: Publications Office. Available at: https://data.europa.eu/doi/10.2776/29620. Last accessed on May 24th, 2022.

[bibr29-02690942221104774] Fluder R Hümbelin O Luchsinger L , et al. (2020) Ein Armutsmonitoring für die Schweiz: Modellvorhaben am Beispiel des Kantons Bern. Schlussbericht. Bern. https://arbor.bfh.ch/id/eprint/12959

[bibr30-02690942221104774] Foulkes M Schafft KA (2010) The impact of migration on poverty concentrations in the United States, 1995-2000: migration on poverty concentrations. Rural Sociology 75(1): 90–110. DOI: 10.1111/j.1549-0831.2009.00002.x 10.1111/j.1549-0831.2009.00002.x

[bibr31-02690942221104774] FSO (2015) Regionalporträts 2015: Gemeinden. Regionalporträts 2015: Gemeinden. Neuchâtel, Switzerland: Federal Statistic Office (FSO).

[bibr32-02690942221104774] FSO (2020) Poverty. Economic and Social Situation of the population. Neuchâtel, Switzerland: Federal Statistical Office (FSO). Available at: https://www.bfs.admin.ch/bfs/de/home/statistiken/wirtschaftliche-soziale-situation-bevoelkerung/wohlbefinden-armut/armut-und-materielle-entbehrungen/armut.html (accessed 4 November 2020).

[bibr33-02690942221104774] Häder S Häder M Kühnex M (2012) Telephone Surveys in Europe. Berlin, Heidelberg: Springer. DOI: 10.1007/978-3-642-25411-6 10.1007/978-3-642-25411-6

[bibr34-02690942221104774] Hainmueller J (2012) Entropy balancing for causal effects: a multivariate reweighting method to produce balanced samples in observational studies. Political Analysis 20(1): 25–46. DOI: 10.1093/pan/mpr025 10.1093/pan/mpr025

[bibr35-02690942221104774] Harrison JL Montgomery CA Jeanty PW (2019) A spatial, simultaneous model of social capital and poverty. Journal of Behavioral and Experimental Economics 78: 183–192. DOI: 10.1016/j.socec.2018.09.001 10.1016/j.socec.2018.09.001

[bibr37-02690942221104774] Hernanz V Malherbet F Pellizzari M (2004) Take-Up of Welfare Benefits in OECD Countries: A Review of the Evidence. OECD Social, Employment and Migration. Working Papers No. 17. Paris, France: OECD Publishing. DOI: 10.1787/525815265414 10.1787/525815265414

[bibr38-02690942221104774] Hotz VJ George R Balzekas J , et al. (1998) Administrative Data for Policy-Relevant Research. Assessment of Current Utility and Recommendations for Development, p. 111.

[bibr39-02690942221104774] Hümbelin O (2019) Non-take-up of social assistance: regional differences and the role of social norms-sciendo. Swiss Journal of Sociology 45(1): 7–33.

[bibr40-02690942221104774] Hümbelin O Farys R (2016) The suitability of tax data to study trends in inequality: a theoretical and empirical review with tax data from Switzerland. Research in Social Stratification and Mobility 44: 136–150. DOI: 10.1016/j.rssm.2016.04.004 10.1016/j.rssm.2016.04.004

[bibr41-02690942221104774] Hümbelin O Fritschi T (2018) Pathways into and out of the labor Market after receiving social benefits: cumulative disadvantage or life course risk? The Sociological Quarterly 59(4): 627–654. DOI: 10.1080/00380253.2018.1489207 10.1080/00380253.2018.1489207

[bibr42-02690942221104774] Hümbelin O Hobi L Fluder R (2021) Rich Cities, Poor Countryside? Social Structure of the Poor and Poverty Risks in Urban and Rural Places in an Affluent Country. An Administrative Data Based Analysis Using Random Forest. Working Papers 40. Bern, Switzerland: University of Bern Social Sciences. Available at: https://ideas.repec.org/p/bss/wpaper/40.html 10.1177/02690942221104774 PMC9253928 35814335

[bibr43-02690942221104774] Katz LF Autor DH (1999) Chapter 26 - changes in the wage structure and earnings inequality. In: Ashenfelter O Card D (ed) Handbook of Labor Economics. Amsterdam, Netherlands: Elsevier. Vol. 3, 1463–1555. DOI: 10.1016/S1573-4463(99)03007-2 10.1016/S1573-4463(99)03007-2

[bibr44-02690942221104774] Katz LF Margo RA (2014) Technical Change and the Relative Demand for Skilled Labor: The United States in Historical Perspective. C12888. National Bureau of Economic Research. DOI: 10.7208/chicago/9780226163925.003.0002 10.7208/chicago/9780226163925.003.0002

[bibr45-02690942221104774] Korinek A Mistiaen JA Ravallion M (2006) Survey nonresponse and the distribution of income. The Journal of Economic Inequality 4(1): 33–55. DOI: 10.1007/s10888-005-1089-4 10.1007/s10888-005-1089-4

[bibr46-02690942221104774] Linder W Zürcher R Bolliger C (2008) Gespaltene Schweiz–geeinte Schweiz: gesellschaftliche Spaltungen und Konkordanz bei den Volksabstimmungen seit 1874. Baden, Switzerland: Hier+ Jetzt, Verlag für Kultur und Geschichte.

[bibr47-02690942221104774] Liu M Hu S Ge Y , et al. (2021) Using multiple linear regression and random forests to identify spatial poverty determinants in rural China. Spatial Statistics 42: 100461. DOI: 10.1016/j.spasta.2020.100461 10.1016/j.spasta.2020.100461

[bibr48-02690942221104774] Lucas B Bonvin J-M Hümbelin O (2021) The non-take-up of health and social benefits: what implications for social citizenship? Swiss Journal of Sociology 47(2): 161–180. DOI: 10.2478/sjs-2021-0013 10.2478/sjs-2021-0013

[bibr49-02690942221104774] Madanipour A (2015) Social exclusion and space. In: The City Reader. Abingdon, UK: Routledge, 237–245.

[bibr50-02690942221104774] Madanipour A Shucksmith M Talbot H (2015) Concepts of poverty and social exclusion in Europe. Local Economy 30(7): 721–741. DOI: 10.1177/0269094215601634 10.1177/0269094215601634

[bibr51-02690942221104774] Madanipour A Weck S (2015) Social exclusion and poverty in Europe: territorial patterns. Local Economy 30(7): 715–720. DOI: 10.1177/0269094215601628 10.1177/0269094215601628

[bibr52-02690942221104774] Michálek A Výbošťok J (2019) Economic growth, inequality and poverty in the EU. Social Indicators Research 141(2): 611–630. DOI: 10.1007/s11205-018-1858-7 10.1007/s11205-018-1858-7

[bibr53-02690942221104774] Moffitt R (1983) An economic model of welfare stigma. The American Economic Review 73(5): 1023–1035.

[bibr54-02690942221104774] Molina M Garip F (2019) Machine learning for sociology. Annual Review of Sociology 45: 27–45.

[bibr55-02690942221104774] Nielsen HS Sørensen T Taber CR (2008) Estimating the Effect of Student Aid on College Enrollment: Evidence from a Government Grant Policy Reform. Working paper. Bonn, Germany: Institute for the Study of Labor (IZA).

[bibr56-02690942221104774] Nosratabadi S Mosavi A Duan P , et al. (2020) Data science in economics: comprehensive review of advanced machine learning and deep learning methods. Mathematics 8(10): 1799. DOI: 10.3390/math8101799 10.3390/math8101799

[bibr57-02690942221104774] O’brien RM (2007) A caution regarding rules of thumb for variance inflation factors. Quality & Quantity 41(5): 673–690. DOI: 10.1007/s11135-006-9018-6 10.1007/s11135-006-9018-6

[bibr58-02690942221104774] Piketty T (2014) Capital in the Twenty-First Century. Cambridge, UK: Harvard University Press.

[bibr59-02690942221104774] R Core Team (2009): R: A Language and Environment for Statistical Computing, R Foundation for Statistical Computing, Vienna, Austria, available at, https://www.r-project.org/. Last accessed on May 24th, 2022.

[bibr60-02690942221104774] Roosma F van Oorschot W Gelissen J (2016) The achilles’ heel of welfare state legitimacy: perceptions of overuse and underuse of social benefits in Europe. Journal of European Public Policy 23(2): 177–196. DOI: 10.1080/13501763.2015.1031157 10.1080/13501763.2015.1031157

[bibr61-02690942221104774] Schweizerische Konferenz für Sozialhilfe (2015) Armut und Armutsgrenze. Grundlagenpapier der SKOS. Bern, Switzerland: SKOS.

[bibr62-02690942221104774] Shucksmith M Schafft K (2012) Rural Poverty and Social Exclusion in the United States and the United Kingdom. Rural Transformations and Rural Policies in the US and UK. Abingdon, UK: Routledge. DOI: 10.4324/9780203144275-15 10.4324/9780203144275-15

[bibr63-02690942221104774] Sidney MS (2009) Poverty, inequality and social exclusion. In: Theories of Urban Politics, 171–187.

[bibr64-02690942221104774] Strobl C Zeileis A (2008) Why and How to Use Random Forest Variable Importance Measures (And How You Shouldn’t).

[bibr65-02690942221104774] Swiss Confederation (2018) Switzerland Implements the 2030 Agenda for Sustainbale Development. Swizterland’s Country Report 2018. Bern, Switzerland: Federal Department of Foreign Affairs FDFA. Available at: https://www.eda.admin.ch/dam/agenda2030/en/documents/laenderbericht-der-schweiz-2018_EN.pdf. Last accessed on May 24th, 2022.

[bibr66-02690942221104774] Tilly C (1999) Durable Inequality. Berkley, Los Angeles, London: University of California Press.

[bibr67-02690942221104774] UN (2017) Guide on Poverty Measurement». New York, Geneva: United Nations.

[bibr68-02690942221104774] Vandecasteele L (2015) Social class, life events and poverty risks in comparative European perspective. International Review of Social Research 5(1): 61–74.

[bibr69-02690942221104774] Wanner P (2019) Vorbereitung einer Datenbank über die wirtschaftliche Situation der Menschen im Arbeits- und Rentenalter. WiSiER, p. 141.

[bibr70-02690942221104774] World Bank (2020) Monitoring Global Poverty. Poverty and Shared Prosperity. Washington, DC: The World Bank. DOI: 10.1596/978-1-4648-1602-4_ch1 10.1596/978-1-4648-1602-4_ch1

[bibr71-02690942221104774] Wright MN Ziegler A (2017) Ranger: a fast implementation of random forests for high dimensional data in C++ and R. Journal of Statistical Software 77(1): 1–17. DOI: 10.18637/jss.v077.i01 10.18637/jss.v077.i01

[bibr72-02690942221104774] Zhou Y Liu Y (2019) The geography of poverty: review and research prospects. Journal of Rural Studies. Epub ahead of print. DOI: 10.1016/j.jrurstud.2019.01.008 10.1016/j.jrurstud.2019.01.008

